# Effects of Nitrogen Nutrition on Accumulation and Speciation of Sulfur Compounds in Garlic Under Field Conditions

**DOI:** 10.3390/plants15172735

**Published:** 2026-09-07

**Authors:** Binh Thi Nguyen, Bernhard J. Wehr, Peter M. Kopittke, Timothy J. O’Hare, Neal W. Menzies, Hung Trieu Hong, Brigid A. McKenna, Wantana Klysubun, Wutthikrai Busayaporn, Stephen M. Harper

**Affiliations:** 1 School of Agriculture and Food Sustainability, The University of Queensland, Gatton, QLD 4343, Australia; b.wehr@uq.edu.au (B.J.W.); p.kopittke@uq.edu.au (P.M.K.); n.menzies@uq.edu.au (N.W.M.); h.trieu@uq.edu.au (H.T.H.); b.mckenna1@uq.edu.au (B.A.M.); 2 Queensland Alliance for Agriculture and Food Innovation, The University of Queensland, Brisbane, QLD 4108, Australia; 3 Synchrotron Light Research Institute, Nakhon Ratchasima 30000, Thailand; wantana@slri.or.th (W.K.); wutthikrai@slri.or.th (W.B.)

**Keywords:** nitrogen management, foliage nutrition, sulfur speciation, nitrogen–sulfur interaction, early diagnostic

## Abstract

Nitrogen (N) supply can influence the accumulation of sulfur (S)-containing metabolites that contribute to garlic (*Allium sativum* L.) flavour and quality, yet evidence of these responses under field conditions remains limited. Nine N application rates (0–360 kg ha^−1^) were evaluated under field conditions to examine the relationships between N supply, plant N and S status, and bulb S-containing metabolites. Allicin was quantified across three varieties, while alliin, γ-glutamyl-S-allyl-L-cysteine (GSAC), and alliin-to-allicin conversion were analysed in Glenlarge. The bulb alliin and allicin concentrations increased linearly with the N rate and were positively associated with the bulb N and S concentrations. The foliage N concentration was strongly positively correlated with the bulb alliin and allicin concentrations, suggesting that leaf N status may provide a useful predictor of S-containing metabolite accumulation. Sulfur K-edge X-ray absorption near-edge structure spectroscopy showed that the relative proportions of GSAC and alliin remained relatively consistent across the six N rates. This indicates that N supply was associated with the increased accumulation of S-containing compounds, without a substantial shift in their relative S speciation. The alliin and allicin concentrations increased up to the highest N rate tested (360 kg N ha^−1^), whereas increases in allicin yield became relatively small above approximately 200 kg N ha^−1^. Overall, these findings provide field-based evidence linking N nutrition with the accumulation of bioactive S-containing compounds in garlic and provide insights into how N supply may influence garlic quality under field conditions.

## 1. Introduction

Garlic (*Allium sativum* L.) is a unique cultivated species having culinary and medicinal properties that result from various S-containing compounds, such as allicin, diallyl sulfide, and various thiols, contributing significantly to its distinctive aroma [1] and health benefits [2,3]. Nitrogen (N), as an essential macronutrient, plays a vital role in plant growth and metabolism, influencing the synthesis of proteins, enzymes, and secondary metabolites. In garlic, N application rates (from 0 to 360 kg ha^−1^) have been evaluated in numerous garlic studies, yet the reported bulb weights vary considerably across experiments, ranging from ~20 to 100 g bulb^−1^ [4,5,6,7,8,9]. However, while there are numerous studies demonstrating the effects of N nutrition on yields (e.g., [4,5,9]), the relationship between the N application rate and the production of S compounds in garlic is still not well understood.

**Figure 1:**
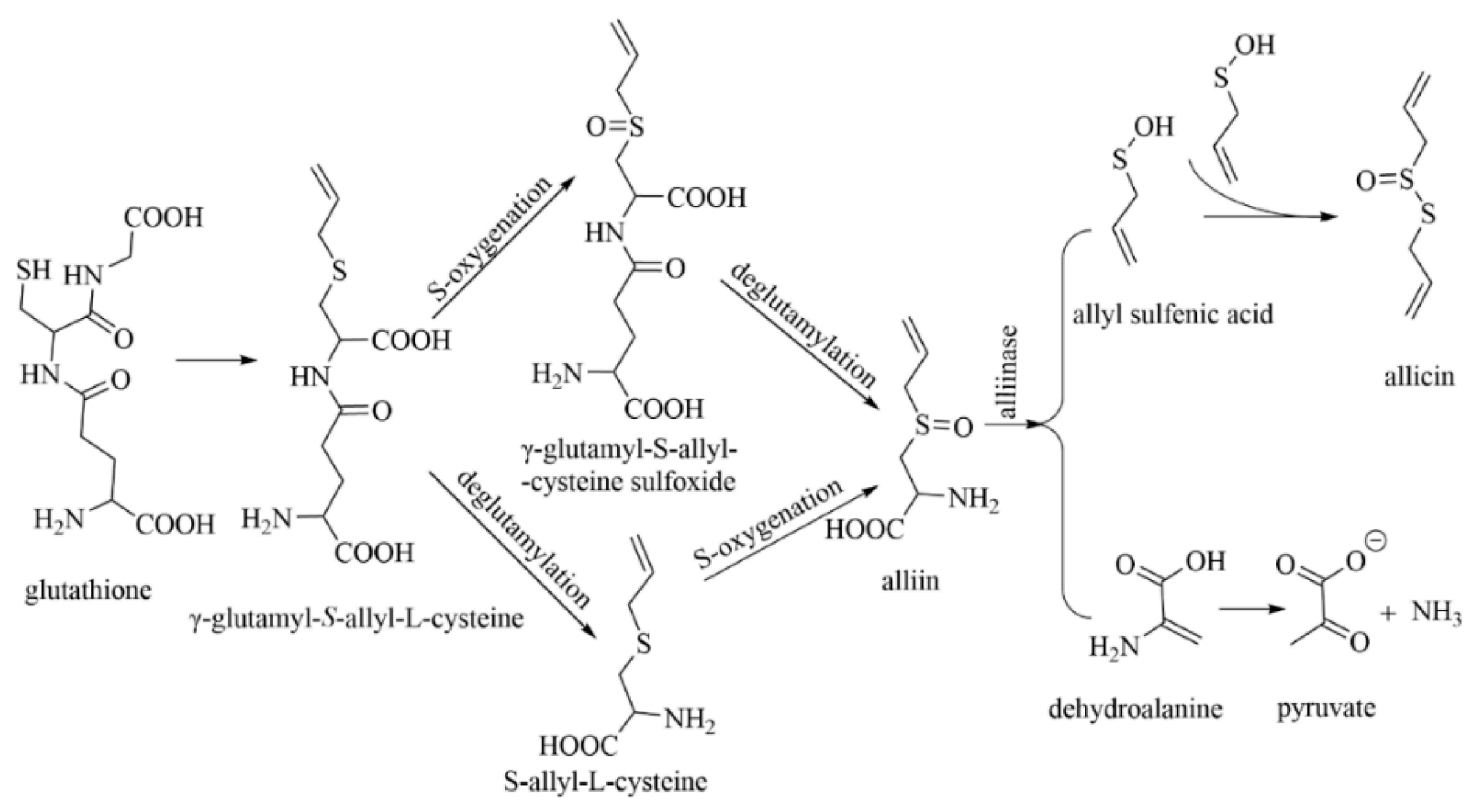
Biosynthesis of alliin, allicin, and other S compounds in garlic [10,11].

In the allicin synthesis pathway (Figure 1), N is a key structural element of allicin’s precursor compounds, such as alliin, γ-glutamyl-S-ally-L-cysteine (GSAC), and glutathione. This biochemical linkage suggests that N might potentially influence not only allicin accumulation but also other S compounds in garlic. However, the extent to which N supply affects the accumulation and partitioning of these compounds, including the conversion of alliin to allicin, remains poorly understood.

A limited number of studies have investigated the effects of N application rates on the quality of garlic in glasshouse trials [12,13] and under field conditions [14]. While pot and solution culture studies provide valuable information under controlled conditions, field-grown plants experience more complex soil–plant nutrient interactions that may influence N and S uptake and metabolism. Importantly, field-based evidence remains limited. Previous studies have primarily quantified alliin [14] or pyruvate [7,15] as proxy indicators of allicin formation, rather than measuring allicin directly. Alliin is the precursor to allicin, and alliin concentrations have been reported to vary widely, ranging from 7.5 to 120 mg g^−1^ [14,16,17]. This variability further highlights the importance of directly quantifying both alliin and allicin when evaluating the effects of N nutrition on garlic S metabolism. Furthermore, whether N supply alters the conversion of alliin to allicin and the partitioning of S among major S-containing compounds under field conditions remains unclear. Conventional metabolite quantification provides information about the concentrations of individual target compounds, whereas S speciation provides complementary information about the distribution of S among different chemical forms. Combining these approaches therefore enables the assessment of whether N supply changes not only the accumulation of S-containing metabolites but also their relative partitioning within the S metabolic pool.

While S nutrition has been shown to affect allicin concentrations [14,18,19], limited information is available regarding how N nutrition influences allicin accumulation. Importantly, the accurate in-season assessment of crop N status is essential for optimising fertiliser management and improving both yields and quality in garlic production systems. Nguyen, Wehr [17] identified strong relationships between the reference leaf mineral status and the bulb yield and allicin concentration across garlic varieties under fixed fertiliser conditions. However, whether such relationships persist under variable N supply remains unknown.

Therefore, this study aimed to investigate the effects of the N application rate under field conditions on (1) the alliin and allicin concentrations and alliin-to-allicin conversion; (2) S speciation and the relative distribution of major S-containing compounds in garlic bulbs; and (3) the relationships between N and S status in foliage and bulbs and the accumulation of alliin and allicin.

## 2. Results and Discussion

### 2.1. Nitrogen Application Rates Affect Alliin and Allicin Concentrations and Allicin Yield

**Figure 2:**
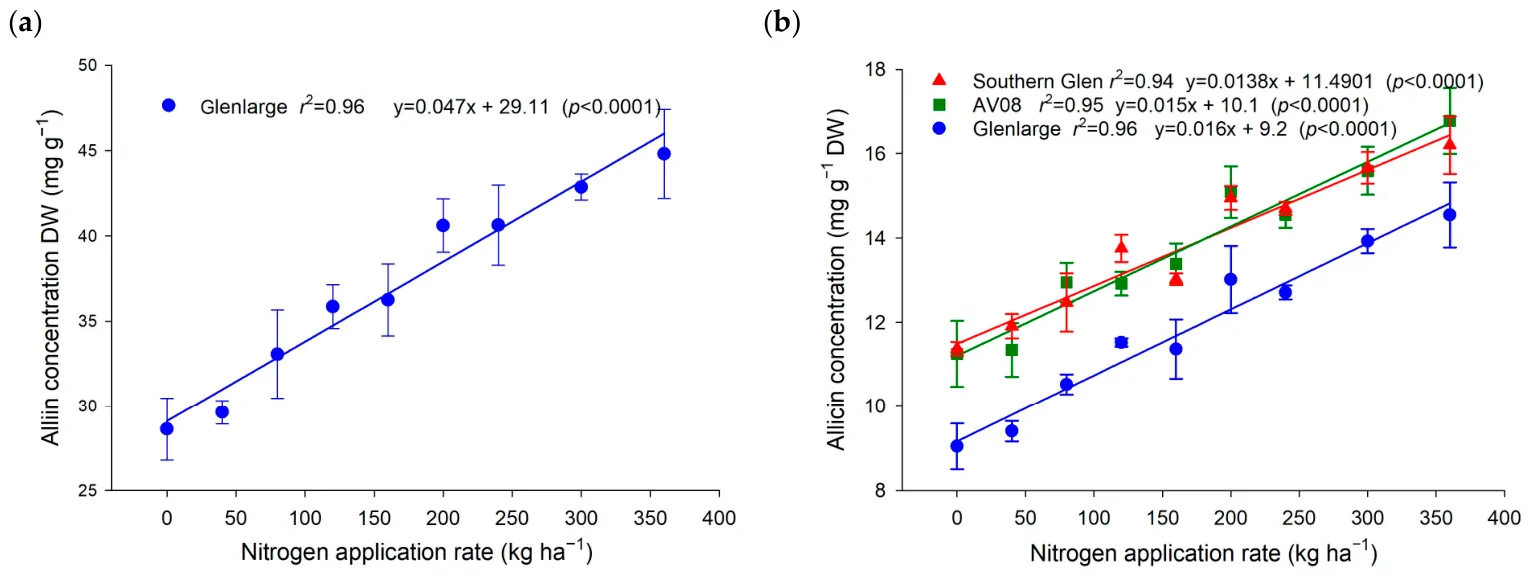
Effects of N application rate on alliin concentration (mg g^−1^ DW) in Glenlarge (**a**) and on allicin concentrations (mg g^1^ DW) across three garlic varieties (**b**). The error bars represent the standard error (*n* = 3).

The alliin concentration of Glenlarge (expressed on a DW basis) increased linearly with the N application rate (r^2^ = 0.96, *p* < 0.0001) (Figure 2a). The alliin concentration was ~28 mg g^−1^ DW in the control (0 kg N ha^−1^) and increased substantially to ~45 mg g^−1^ DW with the application of 360 kg N ha^−1^ (Figure 2a). This is in contrast to various other studies showing that increasing the N rate decreased the alliin concentration in garlic under both glasshouse (pot experiments) [12,13] and field conditions [14]. For example, Bloem, Haneklaus [14] reported a highly fluctuating pattern, with relatively high alliin concentrations at 0 kg N ha^−1^ and a decrease at 50 kg N ha^−1^, followed by an increase at 100 kg N ha^−1^ and a subsequent decline at 150 kg N ha^−1^. This response does not show a consistent dose-dependent relationship with the N application rate, making biological interpretation difficult. Nonetheless, the alliin concentration in the study of Bloem, Haneklaus [14] (~7.4–10.5 mg g^−1^ DW) was substantially lower (about a third) than that in the present study (29–45 mg g^−1^ DW).

In current study, the allicin concentration was strongly positively correlated (r^2^ > 0.94, *p* < 0.0001) with the N application rate across the three varieties (Figure 2b). The split-plot analysis of variance showed significant effects of the N application rate and variety on the allicin concentration (*p* < 0.001), whereas the N application rate × variety interaction was not significant (*p* = 0.995) (Figure S2). Over the range of 0 to 360 kg N ha^−1^, the allicin concentration increased by ~50% for each variety, with that of Glenlarge increasing from ~9 to 13 mg g^−1^ DW and those of both Southern Glen and AV08 increasing from ~11 to 16.5 mg g^−1^ DW (Figure 2b). Across N application rates, the allicin concentration in Glenlarge was consistently lower (by about 2 mg g^−1^ DW) than in Southern Glen and AV08 (Figure 2b and Figure S2). These results indicate that the allicin concentration was significantly influenced by both N supply and the variety.

**Figure 3:**
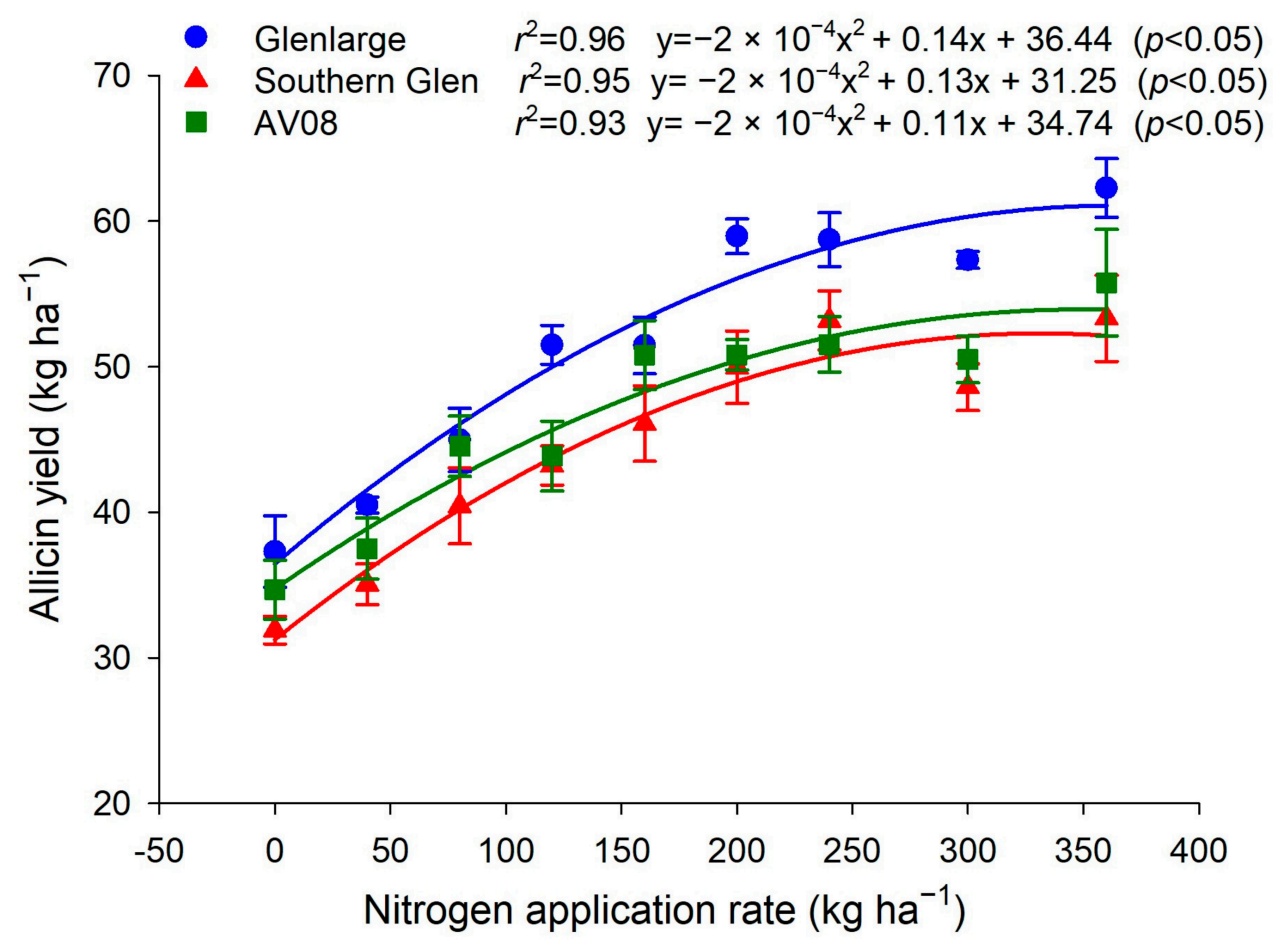
The effects of N application rates (0–360 kg N ha^−1^) on the allicin yield (kg ha^−1^) for three garlic varieties. The error bars represent the standard error (*n* = 3).

The allicin yield was significantly affected by the N application rate and variety (*p* < 0.001), whereas the N application rate × variety interaction was not significant (*p* = 0.758) (Figure S3). As the N application rate increased from 0 to 360 kg ha^−1^, the allicin yield increased by ~67% in Glenlarge (from ~37 to ~62 kg ha^−1^) and by ~65% in Southern Glen and AV08 (~32 to ~53 kg ha^−1^) (Figure 3). Across N application rates, the allicin yield was greater for Glenlarge, while AV08 and Southern Glen had similar allicin yields that were approximately 20% lower than that of Glenlarge (Figure 3 and Figure S3). For all three varieties, increases in allicin yield became relatively small when more than 200 kg N ha^−1^ was applied.

Although the highest alliin and allicin concentrations were observed at 360 kg N ha^−1^, this maximum concentration should not be interpreted as the agronomic optimum N rate. The allicin yield exhibited only marginal increases above approximately 200 kg N ha^−1^, indicating diminishing returns with further N application. This response is consistent with the yield response observed in the same field experiment [9]. Approximately 95–100% of the maximum bulb yield occurred within an N application range of 160–240 kg N ha^−1^, whereas the bulb weight declined at rates above 240 kg N ha^−1^ [9]. Collectively, these findings suggest that applying 360 kg N ha^−1^ solely to maximise the alliin or allicin concentration may provide limited agronomic benefit, particularly when both the bulb yield and allicin yield are considered. From a practical perspective, N management should therefore balance the bulb yield, bioactive compound concentrations, and allicin yield against fertiliser use efficiency and potential environmental impacts. Nevertheless, the increase in alliin and allicin concentrations with N supply may be particularly relevant to the pharmaceutical industry, where garlic with a high allicin concentration is preferred for the manufacture of medicinal products [20].

### 2.2. Effects of N Application Rate on Conversion from Alliin to Allicin

Increasing the N application rate significantly increased the concentrations of both alliin and allicin (*p* < 0.05) (Figure 2), with the highest alliin (0.25 mmol g^−1^ DW) and allicin (0.09 mmol g^−1^ DW) concentrations recorded at the application of 360 kg N ha^−1^ (Table 1). The alliin-to-allicin conversion ratio remained consistent across all treatments, ranging from 2.8 to 2.9, with no significant differences (*p* > 0.05) (Table 1). The alliin-to-allicin conversion ratio was greater than the theoretical value of 2:1, suggesting the inefficiency of the conversion of allyl-sulfenic acid to allicin or the production of other secondary S compounds from allyl-sulfenic acid (e.g., S-methyl prop-2-ene-1-sulfinothioate [21]). This finding is consistent with the study of Nguyen, Wehr [17] in that the stoichiometric alliin-to-allicin conversion ratio for variety Glenlarge was about 2.7. The constant alliin-to-allicin ratio (2.8–2.9) across nine N application rates (from 0 to 360 kg ha^−1^) suggests that, while N fertilisation enhances the biosynthesis of alliin and allicin, the relative relationship between these two compounds remains largely unchanged.

### 2.3. Sulfur Speciation Across Different N Application Rates Using Synchrotron-Based XANES

**Figure 4:**
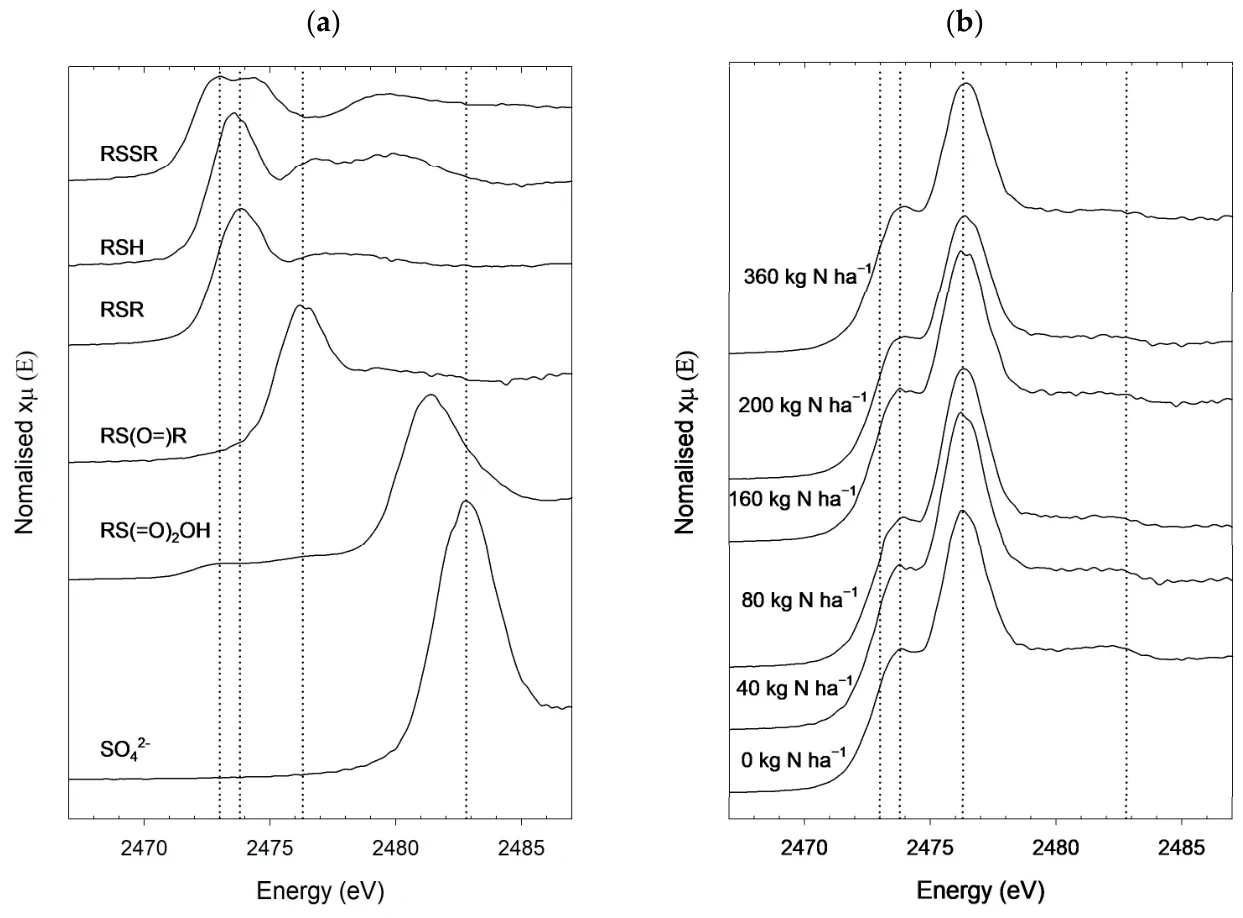
Sulfur K-edge X-ray absorption spectra of (**a**) standard compounds and (**b**) different N application rates. The vertical dotted lines indicate the energies corresponding to the white line peaks of RSSR (2473.0 eV), RSR (2473.7 eV), RS(=O)R (2476.3 eV), and SO_4_^2−^ (2482.8 eV). Top to bottom: RSSR, cystine; RSH, reduced glutathione; RSR, glutamyl-S-allyl-L-cysteine; RS(=O)R, alliin; RS(=O)_2_OH, taurine; SO_4_^2−^, Na sulfate.

Synchrotron-based XANES was used characterise S speciation in garlic. First, the XANES spectra of the various S standard compounds were examined, showing pronounced differences between the different forms of S. The most noticeable differences were in the energy values associated with the white line peak (Figure 4a), which varied primarily according to the oxidation state. Specifically, higher oxidation states corresponded to higher peak energies. For instance, the white line peak was at 2473.6 eV for thiol groups (RSH, oxidation state −2), at 2476.3 eV for sulfoxide groups (RS(=O)R, oxidation state 0, e.g., alliin), and up to 2482.8 eV for sulfate (oxidation state +6). Even closely related compounds showed subtle spectral differences. For example, cysteine (RSH) and methionine (RSR) had white line peaks that differed by ca. 0.1 eV. As expected, disulfide compounds (RSSR, e.g., cystine) displayed two peaks ca. 1.4 eV apart, corresponding to 1s → σ*(S-S) and 1s → σ*(S-C) transitions. Similarly, thiosulfinate compounds (RS(=O)SR, such as allicin) showed two peaks at 2473.0 and 2476.2 eV. In addition, differences in the intensity of the white line peak were also observed. For example, the spectrum of sulfate had a substantially higher white line peak compared to many other S forms. Of the S-containing compounds relevant to garlic, it was observed that the spectrum for alliin [RS(=O)R] had a peak at 2476.3 eV, γ-glutamyl-S-allyl-L-cysteine [RSR] had a peak at 2473.8 eV, and allicin [RS(=O)SR] had two peaks at 2472.5 and 2476.2 eV.

The XANES spectra from the garlic bulbs were then examined to determine the effects of the N application rate on S forms. For this, garlic bulb samples were collected, freeze-dried, and ground into a fine powder. Across samples from six N application rates, two distinct spectral peaks were observed, corresponding to the γ-glutamyl-S-allyl-L-cysteine (GSAC) standard (2473.8 eV, RSR) and the alliin standard (2476.3 eV, RS(=O)R) (Figure 4b). This indicates that these two forms were dominant in garlic bulbs, as reported previously by Nguyen, Wehr [17].

Using LCF, it was predicted that ~44% of the S in the garlic bulbs was present as GSAC compounds and ~56% as alliin across the six N application rates (Table S1). This GSAC-to-alliin proportion differs slightly from that reported by Nguyen, Wehr [17], who found approximately 60% GSAC and 40% alliin. These differences may reflect variations in sample preparation methods, particularly the use of freeze-dried samples in the present study compared with oven-dried samples in Nguyen, Wehr [17]. Notably, although increasing the N application rate resulted in a linear increase in the alliin concentration (Figure 2a), the relative percentages of GSAC and alliin remained relatively consistent across all six N application rates. This suggests that the increasing N supply influenced the overall accumulation of S-containing compounds without substantially altering their relative distributions between GSAC and alliin. Such stability is consistent with the role of GSAC as a precursor of alliin [22]. Together, these findings suggest that N supply may influence the overall accumulation of S-containing compounds without substantially altering the relative proportions of GSAC and alliin.

A limitation of the XANES analysis is that S speciation was assessed for only six of the nine N treatments and was restricted to the Glenlarge variety due to limited synchrotron beamtime. In addition, the three field replicates were combined into a single composite sample for each N treatment; therefore, the observed differences in S speciation could not be subjected to statistical comparison. Further studies incorporating biological replication and additional N treatments and garlic varieties would be valuable to determine the consistency of these responses.

### 2.4. The Overall Role of N in Alliin and Allicin Synthesis

Nitrogen and S are closely associated with the biosynthesis of S-containing compounds in garlic. Nitrogen is a structural component of alliin and several of its biosynthesis precursors, while S is a structural component of both alliin and allicin (Figure 1). In this study, we investigated the effects of nine N application rates on the N and S status in the garlic bulbs and reference leaf of Glenlarge and examined their relationships with the alliin and allicin concentrations.

**Figure 5:**
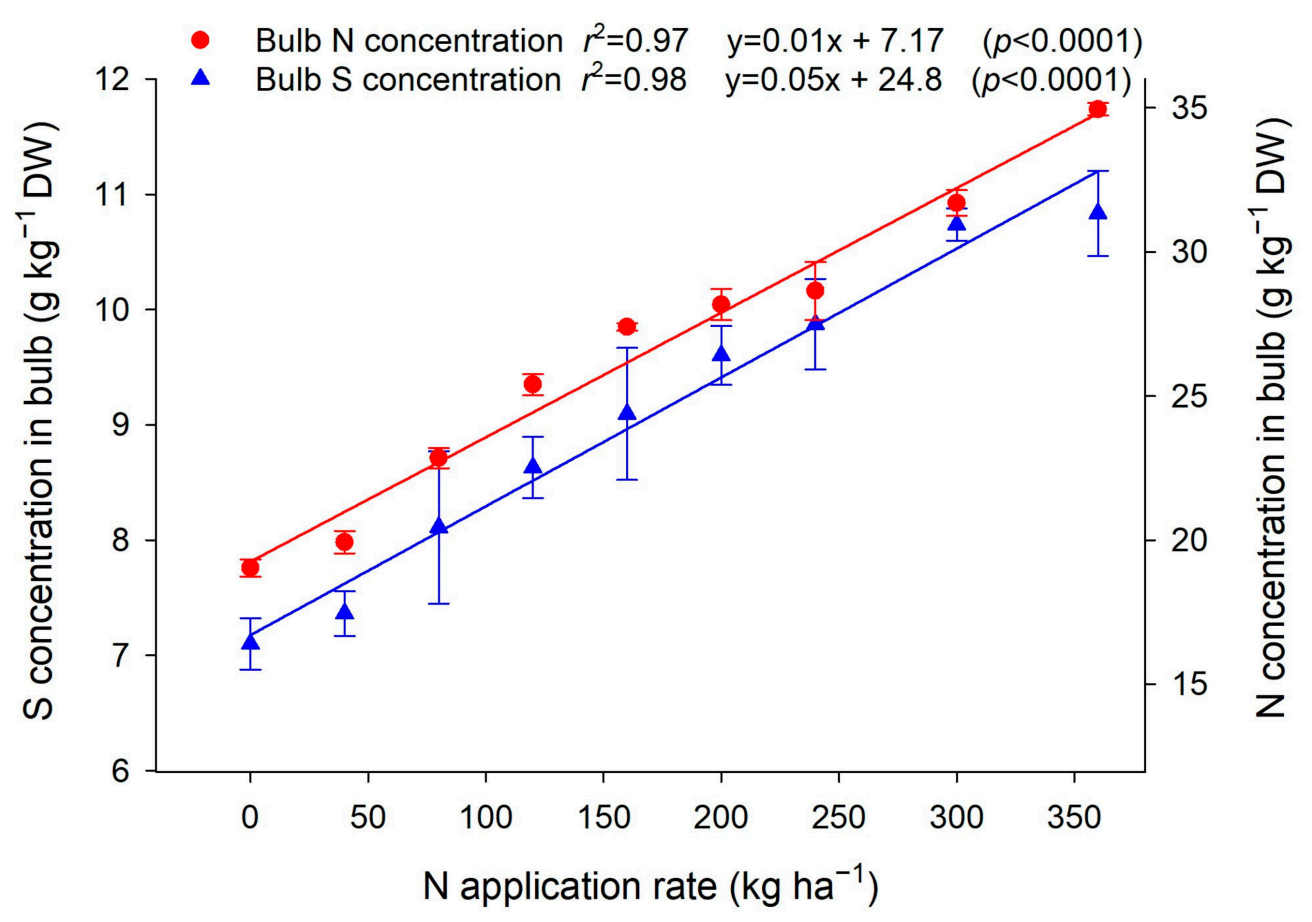
The relationship between the N application rate and N and S concentrations in garlic bulbs of Glenlarge.

**Figure 6:**
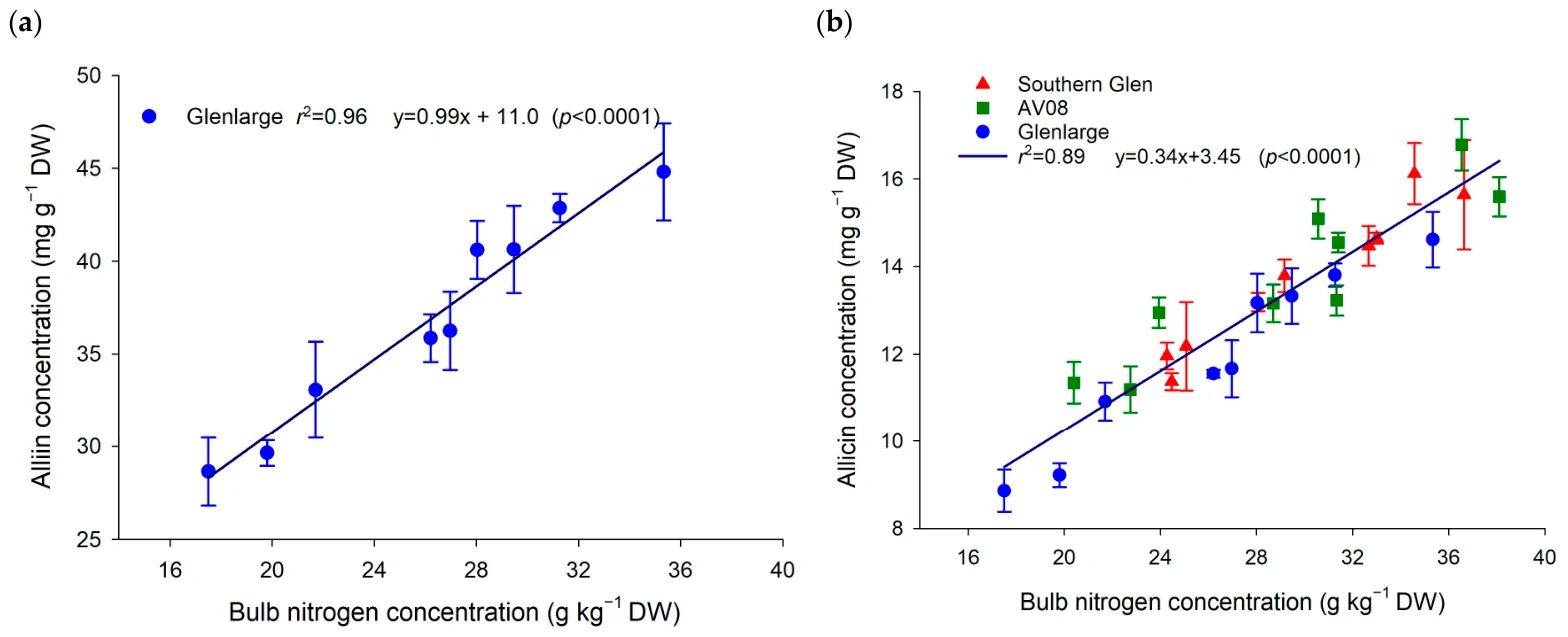
The relationship between the bulb N concentration and the alliin concentration in the Glenlarge variety (**a**) and the allicin concentration in all three varieties (**b**).

**Figure 7:**
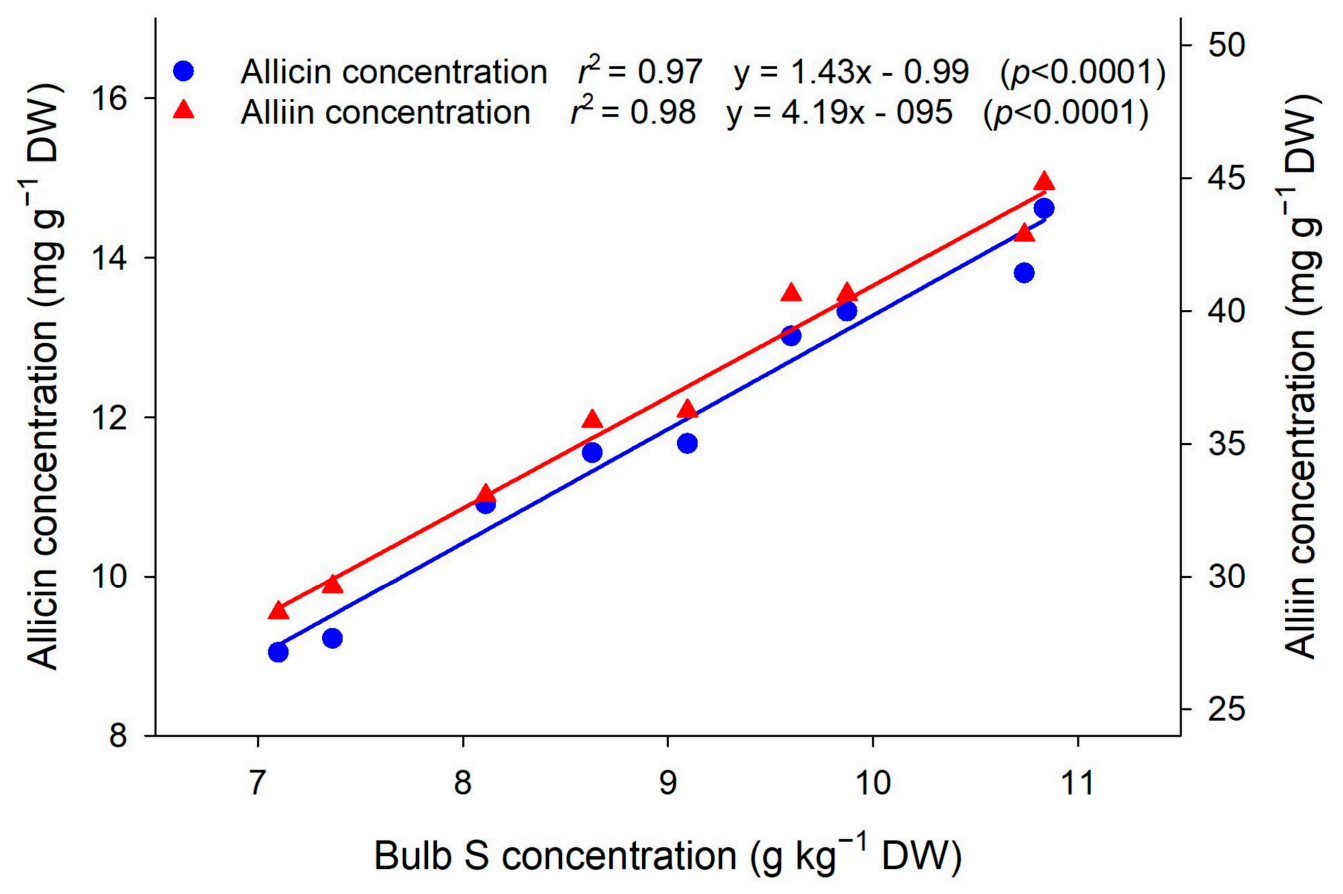
The relationship between the S concentration (mg kg^−1^ DW) in the garlic bulb and the alliin and allicin concentrations (mg g^−1^ DW) in the Glenlarge variety.

In the bulb, we found a linear increase in the N concentration from 20 to 35 g kg^−1^ and in the S concentration from ~7 to 10.5 g kg^−1^ when the N application rate was increased from 0 to 360 kg ha^−1^ (Figure 5). This was accompanied by strong positive relationships between the alliin and allicin concentrations and the bulb N (Figure 6; Table S2) and S concentrations (Figure 7). These findings indicate that a greater plant N status was associated with the increased accumulation of alliin and allicin. This relationship may partly reflect the role of N in the structure of alliin and its biosynthetic precursors (Figure 1). However, the interaction between N and S in alliin biosynthesis may extend beyond their structural roles. Sulfur, taken up as sulfate, is assimilated into cysteine, which contributes to the synthesis of glutathione and γ-glutamyl-cysteine and subsequently to GSAC, a key intermediate in alliin biosynthesis [10]. The conversion of this intermediate to alliin involves deglutamylation by γ-glutamyl transpeptidases followed by S-oxygenation [10]. Thus, the concurrent increases in the bulb N and S concentrations with increasing N supply may reflect coordinated N and S metabolism and the greater allocation of these nutrients to S-containing metabolites such as alliin. However, the activity and expression of the enzymes involved in this pathway were not measured in the present study, and the regulatory mechanisms by which N supply influences alliin biosynthesis require further investigation.

**Figure 8:**
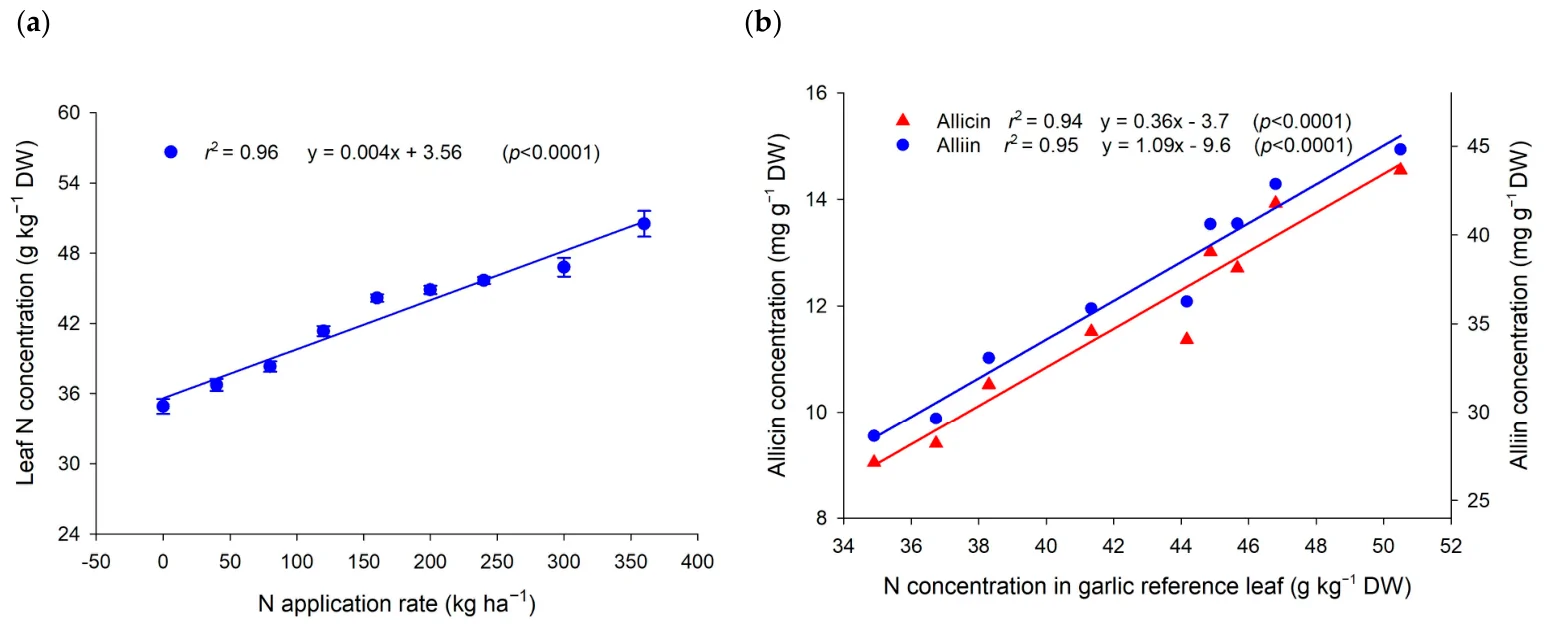
Relationships between reference-leaf concentration and N application rate (0–360 kg ha^−1^) (**a**) and between reference-leaf concentration and allicin and alliin concentrations in Glenlarge bulbs (**b**).

With increasing N application rates, the foliage N concentration increased linearly from ~ 35 to 51 g kg^−1^ (Figure 8a), with strongly positive linear regression with the alliin (r^2^ = 0.95, *p* < 0.0001) and allicin (r^2^ = 0.94; *p* < 0.0001) concentrations in the bulbs (Figure 8b). Importantly, these relationships highlight the strong association between the reference-leaf N status and the subsequent accumulation of alliin and allicin in the bulb and may help to inform N management during crop development.

**Figure 9:**
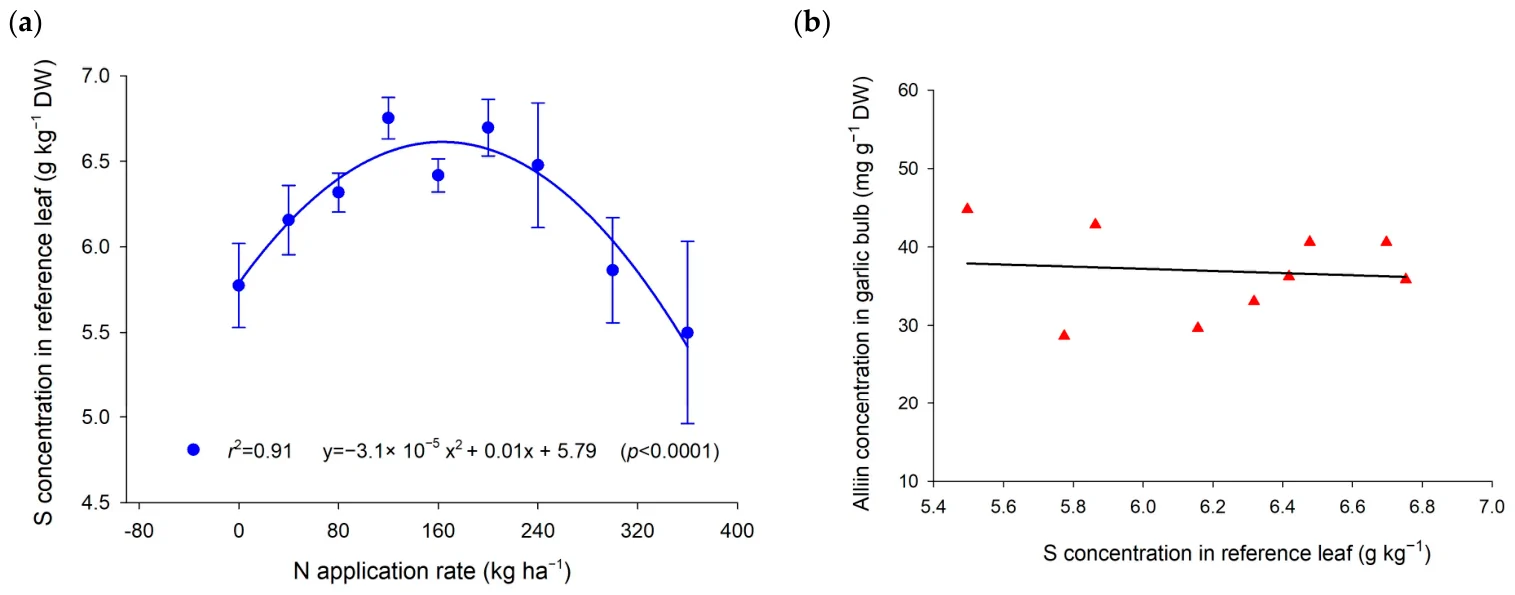
The relationships between the reference-leaf S concentration and N application rate (from 0–360 kg ha^−1^) (**a**) and between the reference-leaf S concentration and bulb alliin concentration (**b**) in Glenlarge. Circles in (**a**) represent treatment means, triangles in (**b**) represent observed values, and lines represent fitted regression models. Error bars in (**a**) represent ± standard error.

In contrast, the N application rate had only a slight effect on the S concentration in the foliage (Figure 9a), and no relationship was observed between the leaf S and bulb alliin concentrations (Figure 9b). Across all N treatments, the foliage S concentrations (~5.5–6.7 g kg^−1^ DW) were maintained within the reported sufficient range (4.80–10.3 g kg^−1^) [17], indicating that S was unlikely to be limiting under these conditions. Although alliin is synthesised in the foliage and then subsequently translocated to the bulb [14], the absence of a relationship between the foliage S concentration and bulb S metabolites suggests that variation in foliage S was not associated with alliin accumulation. By comparison, the strong relationships between the foliage N and bulb alliin and allicin concentrations highlight the potential value of the reference-leaf N status as an indicator of bioactive S-containing compound accumulation.

More broadly, the experiment was conducted during a single growing season at one field location. Therefore, the relationships observed between N supply, plant nutrient status, and S-containing compound accumulation should be validated across additional growing seasons, soil types, and environmental conditions.

## 3. Materials and Methods

A rate trial was established to determine the effects of the N application rate on S compounds in three garlic varieties. The trial was conducted in 2020 at the Queensland Department of Agriculture and Fisheries, Gatton Research Facility, Australia (27°33′ S 152°20′ E). To minimise the soil residual nitrate levels, a barley (*Hordeum vulgare*) cover crop was planted and harvested prior to the garlic trial. Samples from the Dermosol [23] soil were a composite of 20 cores (25–30 cm in depth) randomly collected from each of three blocks, combined, mixed, and subsampled. Soil samples were dried (40 °C for 5 d) and analysed for pH, electrical conductivity (EC), organic carbon (OC), NO_3_^−^, and macro- and micronutrients (Table S3).

The experimental design was a split-plot randomised complete block factorial, with three replications in which the rate of N was the main factor and the varieties were the subfactors. Seed cloves of three garlic varieties differing in allicin concentration, namely Southern Glen (high), Glenlarge, and AV08 (low) [17], were collected from a 2019 field experiment. The dimensions of the main plots were 3.0 m × 5.0 m, consisting of two beds at a 1.5 m width (centre to centre of wheel tracks). Each bed consisted of two rows of garlic at 0.55 m spacing. Within the main plot, two rows were allocated to Glenlarge and one row each to AV08 and Southern Glen. The additional Glenlarge row was included to provide sufficient plant material for the additional chemical and synchrotron analyses conducted on this variety. Glenlarge was selected for detailed alliin analysis, as well the examination of alliin-to-allicin conversion and S speciation, because it is a commonly grown cultivar in the study region. In our previous study, Glenlarge, Southern Glen, and AV08 were classified within the same varietal group based on their morphological and phenotypic characteristics and showed broadly comparable alliin concentrations and alliin-to-allicin ratios [17]. The two Glenlarge rows constituted a single varietal subplot and were not treated as independent experimental units or additional replicates. The buffer at the ends of the plots was 1 m. The within-row plant spacing was 10 cm, giving a plant population density of 133,000 plants ha^−1^.

Nitrogen was applied at rates of 0, 40, 80, 120, 160, 200, 240, 300, and 360 kg ha^−1^, with the timing and proportions applied at each fertigation event provided in Table S4. Urea and Ca(NO_3_)_2_ were chosen as the N sources, with approximately equal amounts of N supplied from each fertiliser source. The soil was inherently well supplied with Ca (12.6 cmolc kg^−1^; Table S3), equivalent to an estimated exchangeable Ca stock of approximately 9.8 t ha^−1^ in the 0–30 cm soil layer. Therefore, the differential supply of Ca through the application of Ca(NO_3_)_2_ was not considered to affect the growth of garlic. Other nutrients were applied at rates considered sufficient to prevent any limitation to crop growth, viz. 130 kg ha^−1^ K, 80 kg ha^−1^ S, 20 kg ha^−1^ Mg, 0.7 kg ha^−1^ B, 0.7 kg ha^−1^ Zn, 0.3 kg ha^−1^ Cu, and 0.11 kg ha^−1^ Mo [17]. The types and amounts of fertiliser and the timing of application are shown in Table S5. Three nutrients, P, Mn, and Fe, were not applied because the soil was inherently high in these compounds. The soil was also inherently high in Mg; however, MgSO_4_ was applied to provide sufficient S.

The trial was irrigated using Rivulis T-Tape 510-20-500 drip irrigation tape (Rivulis Irrigation Ltd., Gvat, Israel) with emitter spacing at 20 cm and an output rate of 1 L h^−1^ per emitter. One line of drip tape was installed on the inside of each row of garlic. Fertiliser was applied through fertigation using a partial flow bypass fertigation tank. Gramoxone was applied prior to planting to control emerged weeds. Irrigation was applied at approximately 25 mm every five days, with the irrigation scheduling adjusted according to the recorded rainfall and pan evaporation (Table S6). Mancozeb, a protectant fungicide, was applied weekly at a rate of 2.2 kg ha^−1^. The trial was thinned to ensure that there were no double plants.

### 3.1. Sample Collection and Processing

The youngest fully mature leaves were collected at the flag leaf stage (about 105 d after planting (DAP)), dried for 7 d at 70 °C, and analysed by inductively coupled plasma optical emission spectroscopy (ICP-OES) for full nutrient analysis. At full maturity (at 185DAP), all remaining plants in each plot were harvested. Five bulbs (55–70 mm diameter) were randomly selected from each plot, and the outer cloves were divided into three subsamples for allicin, alliin, and nutrient analysis. The allicin concentration was analysed in bulbs from all three garlic varieties.

The cloves for allicin analysis were extracted and quantified using the method of Nguyen, Hong [24]. Briefly, the cloves were crushed, and 40 mL of deionized water was added to 1.0 g of the crushed garlic. The extract was centrifuged for 10 min, filtered, and allicin was quantified using a Shimadzu HPLC-PDA (Shimadzu Corporation, Kyoto, Japan) device, as described by Nguyen, Hong [24]. The allicin yield (kg ha^−1^) was calculated by multiplying the bulb allicin concentration (mg g^−1^ DW) by the bulb dry matter yield (t ha^−1^).

The cloves for alliin analysis were prepared, extracted, and quantified following Nguyen, Wehr [17]. Briefly, garlic cloves were immersed in liquid nitrogen and then freeze-dried and ground. Approximately 0.5 g of garlic powder was extracted with 40 mL of deionised water at pH~1, followed by centrifugation for 10 min and filtration. Alliin was quantified using a Shimadzu HPLC-PDA device (Shimadzu, Kyoto, Japan) as described by Nguyen, Wehr [17]. The remaining cloves for nutrient analysis were sliced into 3 cm pieces, dried (70 °C for approximately 5 d), ground, and analysed by a commercial laboratory.

Sulfur compounds were analysed at the S K-edge using X-ray absorption near-edge structure (XANES) spectroscopy, as described by Nguyen, Wehr [17]. Briefly, the measurements were conducted at Beamline 8 of the Synchrotron Light Research Institute (SLRI), Thailand [25]. A double-crystal InSb (111) monochromator was used to select the incident beam energy, with a beam size of ca. 18 × 2 mm. Due to the limited beamtime, garlic bulb samples (variety Glenlarge) were collected from six of the nine N application rates (0, 40, 80, 160, 200, and 360 kg N ha^−1^) for synchrotron analysis. The selected treatments spanned the experimental N gradient from the zero-N control to the highest N application rate. The bulb samples from the selected N treatments were freeze-dried and finely ground using a Retsch MM 500 ball mill (Retsch GmbH, Haan, Germany). For each treatment, the three individual replicates were combined to give a single composite sample for analysis The freeze-dried samples were placed on sulfur-free Kapton tape and analysed in fluorescence mode using a 13-element Ge detector (Ultra-LEGe, Canberra Industries, Meriden, CT, USA), with the sample chamber purged continuously with helium to minimise X-ray absorption by air. In addition, we analysed twelve standard compounds, which are presented to illustrate the different S-bond structures and oxidation states (Table S7). The standards were prepared as described by Nguyen, Wehr [17].

Spectra were recorded across an energy range of 2372 to 2672 eV, with a step size of 5 eV between 2372 and 2462 eV, 0.2 eV between 2462 and 2497 eV, and 5 eV between 2497 and 2462 eV. We used a dwell time of 3 s per energy step for both plant samples and a dwell time of 1.5 s for the reference samples. Three replicate scans were acquired for each of the plant samples, as well as two for the reference samples. All spectra were aligned, normalised, merged, and then analysed using linear combination fitting (LCF) via the Athena Demeter software (v0.9.26; [26]). Representative spectra and fits, including standards, samples, and residuals, are presented in Figure S1.

### 3.2. Statistical Analysis

Data collected across the three garlic varieties were analysed according to the split-plot randomised complete block design, with the N application rate as the main plot factor, the variety as the subplot factor, and three blocks. The nitrogen rate, variety, and their interaction were treated as fixed effects, while the block and the block × N rate interaction were treated as random effects, with the latter providing the main-plot error term. For variables measured only in Glenlarge, the N rate was analysed within a randomised complete block design, with the block included as a random effect. Treatment effects were considered significant at *p* < 0.05. The graphs were generated using the SigmaPlot 14.0 software (Systat Software Inc., San Jose, CA, USA), with error bars representing the standard error of the mean.

## 4. Conclusions

The nitrogen application rate can increase the alliin and allicin concentrations and the allicin yield in garlic bulbs. The alliin and allicin concentrations increased by approximately 40–50% and the allicin yield increased by approximately 65% as the N application rate increased from 0 to 360 kg ha^−1^—the highest N rate tested. However, increases in allicin yield became relatively small above 200 kg N ha^−1^, indicating a diminishing agronomic benefit from further N application. Therefore, the highest N rate tested should not be interpreted as an agronomic optimum, which was not determined in the present study. The XANES analysis of six selected N application rates showed broadly similar relative proportions of the major S-containing compounds across the treatments analysed, although these results were not subjected to statistical comparison. The present study demonstrated positive relationships between the N concentration in the foliage and bulb and the alliin and allicin concentrations. These findings highlight the close relationship between plant N status and the accumulation of these S-containing compounds. In contrast, the N application rate and bulb N concentration had no significant effects on the conversion of alliin to allicin.

## Figures and Tables

**Table 1:** Effects of N application rate (0–360 kg N ha^−1^) on alliin and allicin concentrations and the alliin-to-allicin conversion ratio in garlic bulbs (variety Glenlarge). The alliin-to-allicin conversion ratio was calculated on a molar concentration basis by dividing the alliin concentration by the allicin concentration.

N Treatment (kg ha^−1^)	Alliin Concentration mmol g^−1^ DW	Allicin Concentration mmol g^−1^ DW	Alliin-to-Allicin Conversion Ratio
0	0.16 c	0.056 d	2.9 a
40	0.17 c	0.057 d	2.9 a
80	0.19 bc	0.067 cd	2.8 a
120	0.20 abc	0.071 bcd	2.8 a
160	0.20 abc	0.072 bcd	2.8 a
200	0.23 ab	0.080 abc	2.9 a
240	0.23 ab	0.082 abc	2.8 a
300	0.24 a	0.085 ab	2.8 a
360	0.25 a	0.090 a	2.8 a
*p*-value	<0.001	<0.001	0.987
CV (%)	10.1	8.1	8.8

**Note:** Means followed by different letters within a column are significantly different at *p* < 0.05.

## Data Availability

The data presented in this study are available within the article and its Supplementary Materials.

## Supplementary Materials

The following supporting information can be downloaded at https://www.mdpi.com/article/10.3390/plants15172735/s1. Table S1. Linear combination fitting results for sulfur K-edge XANES spectra of Glenlarge garlic bulbs under six N application rates (0, 40, 80, 160, 200 and 360 kg N ha^−1^); Table S2. Linear regression parameters for the relationships between bulb nitrogen concentration and alliin and allicin concentrations shown in Figure 6. The 95% confidence intervals (CI) are provided for the regression slopes; Table S3. Background analyses of the soil prior to planting; Table S4. Nitrogen fertilisation schedule (g fertiliser per plot (15 m^2^)) including timing of application (days after planting (DAP)), total rate of application (kg N ha^−1^) and fertiliser form (urea or calcium nitrate); Table S5. Application schedule (kg ha^−1^) for the non-N fertiliser; Table S6. The data of temperature (air and soil), relative humidity and sum of rainfall was collected using data logger at Gatton Research Facility in 2020; Table S7. List of standards with different S compound structure and oxidation stage; Figure S1. Linear combination fitting (LCF) of the S K-edge XANES spectrum of Glenlarge garlic bulb at 360 kg N ha-1 using alliin and γ-glutamyl-S-allyl-L-cysteine (GSAC) reference spectra. The measured spectrum, fitted spectrum, individual reference contributions and fitting residual are shown. The R-factor of the LCF was 0.036; Figure S2. Analysis of variance for the effects of nitrogen (N) application rate, garlic variety, and their interaction on bulb allicin concentration. Nitrogen application rate and variety significantly affected allicin concentration (*p* < 0.001), whereas the N application rate × variety interaction was not significant (*p* = 0.995); Figure S3. Analysis of variance for the effects of nitrogen (N) application rate, garlic variety, and their interaction on allicin yield. Nitrogen application rate and variety significantly affected allicin yield (*p* < 0.001), whereas the N application rate × variety interaction was not significant (*p* = 0.758).

## Abbreviations

DAP	Days after planting
DW	Dry weight
GSAC	γ-Glutamyl-S-allyl-L-cysteine
HPLC-PDA	High-performance liquid chromatography with photodiode array detection
HSD	Honest significant difference
ICP-OES	Inductively coupled plasma optical emission spectroscopy
LCF	Linear combination fitting
SLRI	Synchrotron Light Research Institute
XANES	X-ray absorption near-edge structure
