# Supplementary material for: Effects of Nitrogen Nutrition on Accumulation and Speciation of Sulfur Compounds in Garlic Under Field Conditions
Source: Plants (Basel). 2026 Sep 7;15(17):2735. doi: 10.3390/plants15172735 (PMC13568069; doi:10.3390/plants15172735)
Supplement: Supplementary file 1 [file plants-15-02735-s001.zip › plants-4496077-supplementary.pdf]

Effects of Nitrogen Nutrition on Accumulation and Speciation of Sulfur Compounds in Garlic Under Field Conditions

Binh Thi Nguyen [1,*], Bernhard J.Wehr [1], Peter M. Kopittke [1], Timothy J. O'Hare [2], Neal W. Menzies [1], Hung Trieu Hong [1], Brigid A. McKenna [1], Wantana Klysubun [3], Wutthikrai Busayaporn [3] and Stephen M. Harper [1,*]

[1] School of Agriculture and Food Sustainability, The University of Queensland, Gatton, QLD 4343, Australia; b.wehr@uq.edu.au (B.J.W.); p.kopittke@uq.edu.au (P.M.K.); n.menzies@uq.edu.au (N.W.M.); h.trieu@uq.edu.au (H.T.H.); b.mckenna1@uq.edu.au (B.A.M.)

[2] Queensland Alliance for Agriculture and Food Innovation, The University of Queensland, Brisbane, QLD 4108, Australia

[3] Synchrotron Light Research Institute, Nakhon Ratchasima 30000, Thailand; wantana@slri.or.th (W.K.); wutthikrai@slri.or.th (W.B.)

* Correspondence: binh.nguyen@uq.edu.au (B.T.N.); s.harper1@uq.edu.au (S.M.H.); Tel.: +61-440-124-786 (B.T.N.); +61-411-865-100 (S.M.H.)

Supporting information

Table S1. Linear combination fitting results for sulfur K-edge XANES spectra of Glenlarge garlic bulbs under six N application rates (0, 40, 80, 160, 200 and 360 kg N ha^{-1}).

N rate (kg ha^{-1})	Alliin	GASC	R-factor
0	56.8	43.2	0.043
40	56.8	43.2	0.108
80	58.6	41.4	0.067
160	56.1	43.9	0.060
200	55.3	44.7	0.027
360	54.9	45.1	0.036
**Mean**	**56.4**	**43.6**	

Table S2. Linear regression parameters for the relationships between bulb nitrogen concentration and alliin and allicin concentrations shown in Figure 6. The 95% confidence intervals (CI) are provided for the regression slopes.

Figure	Realationship	Regression equation	r^2	*p*-value	95% CI for slope
6a	Bulb N vs alliin	y = 0.99x + 11.07	0.96	<0.0001	0.806–1.164
6b	Bulb N vs allicin	y = 0.34x + 3.45	0.89	<0.0001	0.291–0.389

Table S3. Background analyses of the soil prior to planting.

Soil properties and nutrient	Analytical method	Initial value (mean ± SD)
pH	Aqueous (1:5)	7.92±0.01
EC (dS m^{-1})	Aqueous (1:5)	0.14±0.006
OC (%)	Walkley & Black	1.13±0.02
NO_3^- (mg kg^{-1})	Aqueous (1:5) Flow Injection	11.7±2.5
P (mg kg^{-1})	Colwell	153±5
Cl (mg kg^{-1})	Aqueous (1:5) Flow Injection	33.0±8.5
CEC (cmolc kg^{-1})	CEC alcoholic NH_4Cl pH 8.5 AA	27.7±0.6
Ca (cmolc kg^{-1})	1M ammonium acetate ICP-OES	12.6±0.35
Mg (cmolc kg^{-1})	1M ammonium acetate ICP-OES	12.6±0.57
K (cmolc kg^{-1})	1M ammonium acetate ICP-OES	1.20±0.06
Na (cmolc kg^{-1})	1M ammonium acetate ICP-OES	0.85±0.06
S (mg kg^{-1})	Monocalcium phosphate extraction -ICP-OES	7.0±0.0
Fe (mg kg^{-1})	diethylenetriaminepentaacetic acid -ICP-OES	17.7±1.7
Cu (mg kg^{-1})	diethylenetriaminepentaacetic acid -ICP-OES	4.0±0.4
Zn (mg kg^{-1})	diethylenetriaminepentaacetic acid -ICP-OES	2.1±0.3
Mn (mg kg^{-1})	diethylenetriaminepentaacetic acid -ICP-OES	31.9±5.2

Table S4. Nitrogen fertilisation schedule (g fertiliser per plot (15 m^2)) including timing of application (days after planting (DAP)), total rate of application (kg N ha^{-1}) and fertiliser form (urea or calcium nitrate).

Treatments	10 DAP		30 DAP		50 DAP		70 DAP		90 DAP	
(N target (kg ha^{-1})	Urea	$Ca(NO_3)_2$	Urea	$Ca(NO_3)_2$	Urea	$Ca(NO_3)_2$	Urea	$Ca(NO_3)_2$	Urea	$Ca(NO_3)_2$
0	0	0	0	0	0	0	0	0	0	0
40	65.3	193.5	0	0	0	0	0	0	0	0
80	65.3	193.5	65.3	193.5	0	0	0	0	0	0
120	65.3	193.5	65.3	193.5	65.3	193.5	0	0	0	0
160	65.3	193.5	65.3	193.5	65.3	193.5	65.3	193.5	0	0
200	65.3	193.5	65.3	193.5	65.3	193.5	65.3	193.5	65.3	193.5
240	65.3	193.5	65.3	193.5	130.5	387.0	65.3	193.5	65.3	193.5
300	65.3	193.5	65.3	193.5	130.5	387.0	130.5	387.0	130.5	387.0
360	65.3	193.5	65.3	193.5	130.5	387.0	130.5	387.0	195.8	580.8

Table S5. Application schedule (kg ha^{-1}) for the non-N fertiliser.

Fertiliser	Total rate applied (kg ha^{-1})	Increment application (kg ha^{-1})		
		10 DAP	30 DAP	60 DAP
K_2SO_4	290	86.7	116	87
$MgSO_4.7H_2O$	204	61.2	81.6	61.2
$ZnSO_4. 7H_2O$	3.08	0.92	1.23	0.92
$Na_2B_8O_{13}·4H_2O$	3.34	1.00	1.34	1.00
$CuSO_4.5H_2O$	1.18	0.35	0.47	0.35
$Na_2MoO_4.2H_2O$	0.277	0.083	0.113	0.083

Table S6. The data of temperature (air and soil), relative humidity and sum of rainfall was collected using data logger at Gatton Research Facility in 2020.

Month	Air Temperature			Soil temperature			Relative humidity			Rain
	Mean	Max	Min	Mean	Min	Max	Mean	Min	Max	
Apr	20.7	23.7	19.1	30.7	10.7	31.0	64.0	0.0	100	64.0
May	17.1	19.5	12.8	25.5	3.9	28.5	53.4	0.0	100	70.2
Jun	14.4	16.3	11.7	21.3	2.3	27.9	57.3	0.0	100	11.4
Jul	14.4	16.2	11.3	20.6	0.6	27.5	56.2	0.0	100	3.8
Aug	15.0	16.4	12.0	20.6	2.3	28.9	55.3	0.0	100	2.0
Sep	18.9	20.4	14.9	27.0	4.2	35.8	52.7	0.0	100	0.2
Oct	21.5	23.9	19.5	27.7	7.6	41.1	58.2	0.0	100	0.4
**Mean/ total**	**18.4**	**20.4**	**15.1**	**25.9**	**5.6**	**32.8**	**57.5**	**0.0**	**100**	**152**

Table S7. List of standards with different S compound structure and oxidation stage

Standard compound	Structure	Oxidation state	Functional group
L-Glutathione Reduced	R-S-H	-2	Thiol
D-Cysteine	R-S-H	-2	Thiol
L-Methionine	R-S-R	-2	Thio-ether
Glutamyl-S-allyl-L-cysteine (GSAC)	R-S-R	-2	Thio-ether
Cystine	R-S-S-R	-1	Disulfide
Allicin	R-S(=O)-S-R	1 and +1	Thio-sulfinate
Alliin	R-S(=O)-R	0	Sulfoxide
Hypo-taurine	RSO(OH)	+2	Sulfinic acids
Taurine	$RS(=O)_2OH$	+4	Sulfonic acids
Sodium sulfate	Na_2SO_4	+6	Free sulfate

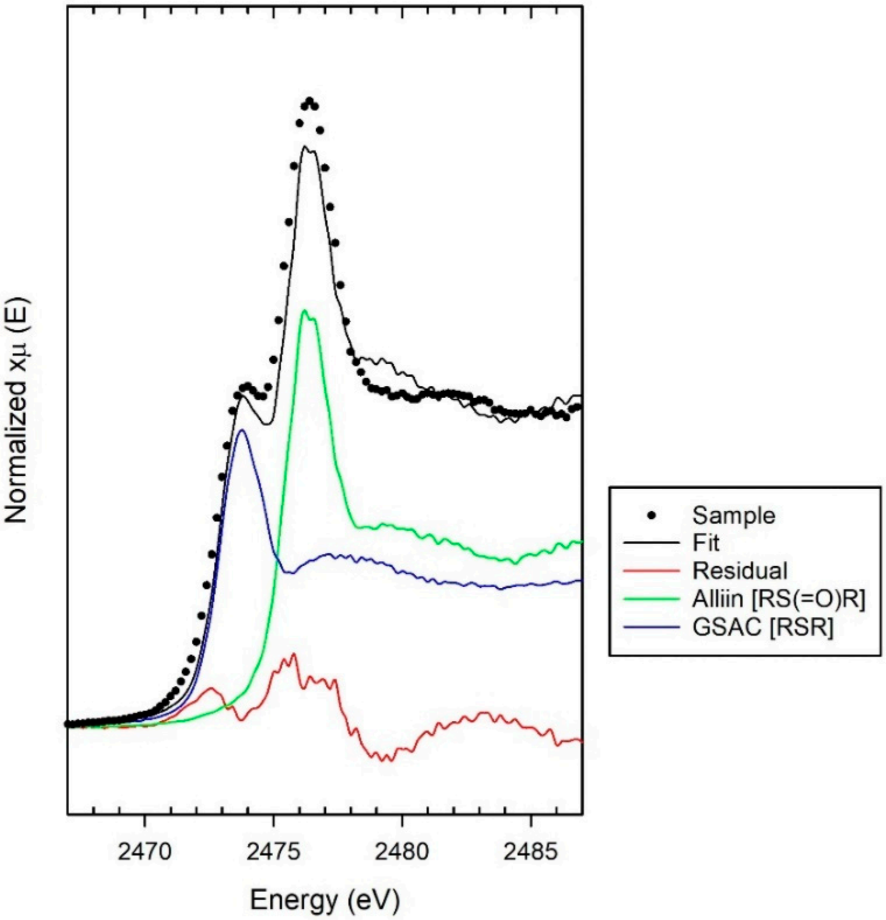

Figure S1. Linear combination fitting (LCF) of the S K-edge XANES spectrum of Glenlarge garlic bulb at 360 kg N ha^{-1} ^{ss}using alliin and γ-glutamyl-S-allyl-L-cysteine (GSAC) reference spectra. The measured spectrum, fitted spectrum, individual reference contributions and fitting residual are shown. The R-factor of the LCF was 0.036.

Analysis of variance

Variate: Allic_Conc_DW

Source of variation	d.f.	s.s.	m.s.	v.r.	F pr.
Rep stratum	2	14.3779	7.1889	9.00	
Rep.N_Treat stratum.					
N_Treat	8	241.0387	30.1298	37.71	<.001
Residual	16	12.7840	0.7990	1.11	
Rep.N_Treat.Variety stratum.					
Variety	2	71.2441	35.6220	49.35	<.001
N_Treat.Variety	16	3.2823	0.2051	0.28	0.995
Residual	36	25.9861	0.7218		
Total	80	368.7130			

Figure S2. Analysis of variance for the effects of nitrogen (N) application rate, garlic variety, and their interaction on bulb allicin concentration. Nitrogen application rate and variety significantly affected allicin concentration ($p < 0.001$), whereas the N application rate × variety interaction was not significant ($p = 0.995$).

Analysis of variance

Variate: Allicin Yield

Source of variation	d.f.	s.s.	m.s.	v.r.	F pr.
Rep stratum	2	74.05	37.02	2.66	
Rep.N_Treat stratum.					
N_Treat	8	4331.60	541.45	38.90	<.001
Residual	16	222.73	13.92	1.20	
Rep.N_Treat.Variety stratum.					
Variety	2	659.64	329.82	28.45	<.001
N_Treat.Variety	16	133.12	8.32	0.72	0.758
Residual	36	417.34	11.59		
Total	80	5838.49			

Figure S3. Analysis of variance for the effects of nitrogen (N) application rate, garlic variety, and their interaction on allicin yield. Nitrogen application rate and variety significantly affected allicin yield ($p < 0.001$), whereas the N application rate × variety interaction was not significant ($p = 0.758$)
